# Evaluation of Surgically Treated Primary Spinal Cord Tumors in a Single Brazilian Institution: A Case Series Study of 104 Patients

**DOI:** 10.7759/cureus.23408

**Published:** 2022-03-22

**Authors:** Eustáquio C Santos Júnior, François Dantas, Antônio Carlos V Caires, Gustavo A Cariri, Marco Túlio D Reis, Ricardo V Botelho, Fernando Luiz R Dantas

**Affiliations:** 1 Neurological Surgery, Biocor Instituto, Belo Horizonte, BRA; 2 Neurological Surgery, Instituto de Assistência Médica ao Servidor Público Estadual (IAMSPE), São Paulo, BRA

**Keywords:** epidemiology, extradural, extramedullary, intramedullary, intradural, tumor, spine, spinal cord

## Abstract

Background: Primary spinal cord tumors are rare and heterogeneous, and their prevalence varies among the studies. Few articles have evaluated the prevalence, characteristics, and histological types of spinal cord tumors in Latin American populations. This study aimed to analyze the histological types and clinical aspects of a series of consecutive patients diagnosed with primary spinal cord tumors who underwent surgical treatment in a single Brazilian institution and to compare them with the literature.

Methods: This is a case series study, with retrospective analysis of all consecutive adult patients who underwent surgical treatment for primary spinal cord tumors in a single center between January 1997 and April 2021. Data analyzed included age at surgery, sex, anatomical location, histopathological diagnosis, clinical presentation, and neurological status at discharge.

Results: A total of 104 patients (53 women [51.0%]; mean age, 49.0 ± 16.7 years [range, 19-87 years]) were included in the analysis. Among the tumors, 83.7% were benign, and 36.5% involved the thoracic spine; intradural extramedullary lesions comprised 52.9% of the tumors, and the most prevalent were schwannomas (26.9%) and meningiomas (18.3%). Among the patients, 55% and 50% presented with pain and motor deficit, respectively, and the deficit improvement rate was greater than the worsening rate at the immediate postoperative period and discharge.

Conclusions: Our series highlights the heterogeneity of primary spinal cord tumors compared to other studies. Further large population studies are necessary to elucidate the epidemiology of this disease.

## Introduction

Primary spinal cord tumors are rare, corresponding to 2%-4% of all central nervous system tumors [1,2]. In addition to being rare, these tumors are very heterogeneous, and there are several possible histological types, such as ependymomas, astrocytomas, schwannomas, and neurofibromas [2]. 78% are benign [3], and they may occupy different compartments of the spinal canal (extradural, intradural extramedullary, and intramedullary) in different segments of the spine [4]. Treatment is usually challenging, and surgery is still the mainstay treatment for many of these lesions [5].

Epidemiological indicators, such as the incidence and prevalence of these lesions, seem to vary in geographically and ethnically different populations [6], and most studies have been conducted in the USA, Europe, and Asia [7]. There are few articles regarding the epidemiology of these lesions in Latin American populations. Therefore, this study aimed to analyze the histological types and clinical aspects of all consecutive adult patients diagnosed with primary spinal cord tumors who underwent surgical treatment in a single institution and compare this series with the literature.

## Materials and methods

We retrospectively analyzed all consecutive patients diagnosed with primary spinal cord tumors who underwent surgical treatment in a single private institution in Brazil between January 1997 and April 2021. The patients were operated on by four different surgeons in the neurosurgical department (FLRD, ACVC, GAC, and MTDSR), and all histopathological examinations were performed by the same pathologist. From 2005 onwards, all surgeries were performed with intraoperative neurophysiological monitoring.

The inclusion criteria were as follows: age > 18 years and diagnostic hypothesis of a primary spinal cord tumor. The exclusion criteria were as follows: (1) histopathological diagnosis incompatible with a primary spinal cord tumor, (2) incomplete medical records, (3) patients who had previously undergone surgery at another institution (in cases in which medical records or histopathological analyses were unavailable), (4) craniocervical junction tumors, and (5) primary spinal bone tumors.

To assess anatomical aspects of the tumors, the location of the lesions was evaluated in two ways: (1) spinal level and (2) spinal compartment. The first concerns the location of the lesion in the neuroaxis, which can be cervical, cervicothoracic, thoracic, thoracolumbar, lumbar, or lumbosacral. The second refers to the location of the lesion in the extradural, intradural extramedullary, or intramedullary space, which may also be of mixed location (intra-and extramedullary, or dumbbell).

The neurological examination of the patients was analyzed at admission and at hospital discharge. This information was synthesized into the following groups of symptoms: “motor deficit,” “sensory deficit,” “pain,” and “sphincter disorders.” Quantitative variables were identified as “present” or “absent” at admission, and “improvement,” “worsening,” “new deficit,” or “unchanged” at discharge.

Data analysis was performed using absolute numbers and percentages. This study was approved by the Biocor Instituto's Research Ethics Committee.

## Results

From 148 patients with a diagnostic hypothesis of a primary spinal cord tumor who underwent surgical treatment, 10 were aged <18 years at the time of surgery, 17 had incomplete medical records, and 17 had a retrospective histopathological diagnosis incompatible with a primary spinal lesion (seven metastases and 10 exams with nonspecific or inconclusive findings). In total, 104 patients were included in the final analysis.

Among the included patients, the mean age was 49 ± 16.7 years (range, 19-87 years), and 49% were aged 45-65 years at the time of surgery. There was an almost equal distribution between men (49%) and women (51%), in addition to a greater proportion of benign over malignant lesions (83.7% vs. 16.3%, respectively). Regarding the location of lesions by spinal segment, there was a preponderance of thoracic tumors (36.5%); however, if we combined all lesions with some extension to the lumbar spine (thoracolumbar, lumbar, and lumbosacral segments) in the same group, we obtained a very close number (37.5%). Regarding the spinal compartment in which the tumors were found, 52.9% of the samples were intradural and extramedullary (Table 1).

**Table 1:** Demographics, surgical characteristics, and clinical features

	n (N=104)	Percentage
Sex		
Female	53	51.0%
Male	51	49.0%
Age (years)		
18-20	2	1.92%
21-40	34	32.7%
41-60	38	36.5%
>60	30	28.8%
Benign/Malignant		
Benign	87	83.7%
Malignant	17	16.3%
Spinal segment		
Thoracic	38	36.5%
Lumbar	25	24.0%
Cervical	23	22.1%
Lumbosacral	11	10.6%
Cervicothoracic	4	3.8%
Thoracolumbar	3	2.9%
Spinal compartment		
Intradural extramedullary	55	52.9%
Intramedullary	24	23.1%
Extradural	18	17.3%
Dumbbell	7	6.7%
	n (N=100)	Percentage
Presentation		
Pain	55	55.0%
Motor deficit	50	50.0%
Sensory deficit	27	27.0%
Sphincter disorders	18	18.0%

The most common histological type was schwannoma (26.9%), followed by meningioma (18.3%), and ependymoma (13.5%). 15.4% of the sample had lesions classified as “others”; these were lesions that did not recur in the sample, such as neuroenteric cyst and germinoma (Table 2). Schwannoma was the most common histological type among dumbbell-shaped tumors (85.7%) and intradural extramedullary tumors (38.2%). The most common intramedullary tumor was ependymoma (41.7%), while other histological types of tumors corresponded to 61.1% of extradural lesions.

**Table 2:** Primary spinal cord tumors based on pathology

Type of tumor	N	Percentage
Schwannoma	28	26.9%
Meningioma	19	18.3%
Ependymoma	14	13.5%
Astrocytoma	6	5.8%
Hemangioblastoma	6	5.8%
Sarcoma	4	3.8%
Cavernoma	3	2.9%
Arachnoid cyst	2	1.9%
Ganglioneuroma	2	1.9%
Neurofibroma	2	1.9%
Malignant peripheral nerve sheet tumor	2	1.9%
Others	16	15.4%

A total of 100 medical records provided satisfactory information about the neurological examination at admission. The most frequently altered domain was pain (including axial pain such as neck pain or low back pain, as well as radicular or neuropathic pattern pain) (55%), followed by motor deficits (50%) (Table 1). Clinical data at admission were compared with those at hospital discharge in 74 of the analyzed records. Events that were “present” at admission were evaluated as “improvement,” “worsening,” or “unchanged” at discharge. Events that were “absent” at admission were assessed as “unchanged” or “new deficit” at discharge (Table 3). Interestingly, in all events, the rate of improvement exceeded the rate of worsening at discharge.

**Table 3:** Comparison between neurological status at admission and at discharge

Neurological status at admission		n (N=74) (%)	Neurological status at discharge			
			New deficit	Unchanged	Improvement	Worsening
Motor deficit	Present	38 (51.4%)	0 (0%)	19 (25.7%)	13 (17.6%)	6 (8.1%)
	Absent	36 (48.6%)	4 (5.4%)	32 (43.2%)	0 (0%)	0 (0%)
Sensory deficit	Present	25 (33.8%)	0 (0%)	17 (23%)	8 (10.8%)	0 (0%)
	Absent	49 (66.2%)	4 (5.4%)	45 (60.8%)	0 (0%)	0 (0%)
Pain	Present	37 (50%)	0 (0%)	8 (10.8%)	29 (39.2%)	0 (0%)
	Absent	37 (50%)	0 (0%)	37 (50%)	0 (0%)	0 (0%)
Sphincter disorders	Present	13 (17.6%)	0 (0%)	8 (10.8%)	5 (6.8%)	0 (0%)
	Absent	61 (82.4%)	1 (1.4%)	60 (81.1%)	0 (0%)	0 (0%)

In patients who presented with pain, there was a 39.2% rate of improvement against 0% of patients who worsened or had a new pain complaint, showing that pain improved better than the other symptoms. In the case of motor deficit, the difference was smaller than in other events: 17.6% of improvement versus 13.5% of worsening or new deficit.

## Discussion

The epidemiology of primary spinal cord tumors varies in geographically and ethnically different populations [6], and there is a gap in the literature regarding the knowledge of this pathology in Latin American populations. Similar to other studies [8-10], we sought to compile all primary spinal cord tumors surgically treated in a single institution to analyze the characteristics of this population.

Our series included 104 primary spinal cord tumors operated in a single center, selecting only adult patients, and excluding metastatic lesions. After a non-systematized literature review, studies that used the same inclusion and exclusion criteria in a similar geographic context were scarce. Despite the heterogeneity, there are several series of cases and database studies of primary spinal cord tumors (Table 4).

The mean age of patients in our series was 49 years, close to the upper limit of the variation found in similar studies (range, 26-55.4 years) [11,12]. It is interesting to note that almost all studies included children in their sample.

In addition to having selected only adult patients, another unusual feature of our series is its geographic location. We found only four Brazilian case series on the subject: two referring only to intramedullary tumors [13,14], one referring only to intradural and extramedullary tumors [15], and one contemplating primary and secondary spinal tumors in both compartments [16].

In other Latin American countries, three case series were found, but all with small samples. A Cuban study gathered 20 primary lesions [17]; two slightly more robust studies were Mexican, with 35 [10] and 27 patients [18], the latter with only intradural and extramedullary lesions.

Most publications regarding primary spinal cord tumors were from the United States and Asian countries and had larger sample sizes. Using more comprehensive databases, such as the National Program of Cancer Registries (NPCR) and Surveillance, Epidemiology, and End Results (SEER), one study managed to include thousands of patients, with representativeness between 89.4% and 99.2% of the North American population in a given period [3]. The most robust Asian studies also included patients from several hospitals to compose the sample [19]. Only three series and our own managed to gather >100 patients in a single center [11,12,20].

In our series, the proportion of benign and malignant lesions (83.7% vs. 16.3%) was quite similar to that described in the largest series on the subject (78% vs. 22%) [3].

Regarding sex distribution, we found an almost equal rate between men (49%) and women (51%). In other Brazilian publications [13,15] and in most Asian articles, the proportion of men was larger, reaching 70.2% of the sample in an Indian series [11]. In the US and European studies, women accounted for approximately 59% of cases [3,21].

Among other case series that included patients with primary spinal cord tumors, both intramedullary and extramedullary, the most frequent histological type in Asian studies was schwannoma. This proportion seems to be especially increased in the Japanese population, where schwannomas account for 56% and 57.2% of the lesions [7,19]. This trend seems to be repeated in the Latin American population, being the most frequent histological type in a Mexican series [10] and in our sample. In contrast, all North American series had meningioma as the most frequent histological type, accounting for 24.4%-42.8% of samples [21,22]. This trend is also observed in a Spanish study [20].

Analysis of case series of only intradural and extramedullary tumors showed schwannoma as the most frequent histological type in most studies, including a Brazilian series [15] and a South African series [23].

Regarding intramedullary tumors, the most frequent histological type in our series was ependymoma, which is in line with the findings of all other analyzed series, except for a study from Singapore, in which this histological type was as frequent as astrocytomas [8].

Regarding the most affected spinal segment, our finding (thoracic segment in 36.5% of cases) was corroborated by what has been described in other studies, in which this location accounts for approximately 40% of the lesions [8-10].

As for the patients' initial clinical presentation, the most common symptoms in our series were pain (55%) and motor deficits (50%), followed by sensory deficits (27%) and sphincter disorders (18%). In other case series and literature reviews, the pain was also the most frequent symptom, in 51%-72% of patients, followed by motor deficits (36%-55%), sensory deficits (12%-39%), and sphincter disorders (15%) [1,9].

Regarding the clinical evolution of these patients, we found an improvement rate between 6.8% and 39.2%, with stability rates between 10.8% and 81.1%, and worsening and new deficit rates between 1.4% and 13.5%. These values ​​were obtained stratified by groups of symptoms, while most studies in the literature provided data on the overall rate of improvement or worsening, which makes a direct comparison limited. In this case, the values ​​vary between 43% and 62.5% of general improvement and 1.1%-22.9% of worsening [8,13,15].

Several factors still make it difficult to compare outcomes from different series. For example, there was great heterogeneity in the way symptoms were classified and quantified. Different scales have been used in different studies, the most frequent being those by McCormick, Klekamp, Samii, and Frankel [5], or even the classification of the American Spinal Injury Association [24]. In addition, the timing of the assessment varied significantly between the studies, with those with a follow-up of days to months [24,25], in contrast to other studies that assessed the clinical picture at discharge, as in our series. Despite the heterogeneity described, both in our series and in other studies analyzed, the clinical stability rate exceeded the rate of improvement, which was higher than the rate of worsening or new deficit.

**Table 4:** Case series and data-base studies of primary spinal cord tumors Abbreviations: IDEM, intradural extramedullary; IMSCT, intramedullary spinal cord tumor; N/A, not available *Case series that included only intradural extramedullary tumors ^+^Case series that included only intramedullary tumors

Author & Year	Country	Year	N	% IDEM (most common)	% IMSCT (most common)	Female/Male	Mean age (years)
Preston-Martin, 1990 [22]	USA	1972–1985	462	N/A	N/A	59.5%/40.5%	N/A
Hufana et al., 2005[8]	Singapore	1992–2002	93	79.6% (schwannoma)	9.7% (astrocytoma / ependymoma)	52.7%/47.3%	49.0
Gelabert-González, 2007 [20]	Spain	1980–2004	168	66.1% (meningioma)	33.9% (ependymoma)	58.3%/41.7%	N/A
Schellinger et al., 2008 [6]	USA	1998–2002	3,226	N/A	N/A	55%/45%	51.0
Alpízar-Aguirre et al., 2009 [18] *	Mexico	1996–2006	27	100% (schwannoma / meningioma)	0%	40.7%/59.3%	47.3
Engelhard et al., 2010 [21]	USA	2000	430	N/A	N/A	56.7%/43.3%	49.3
Hirano et al., 2012 [19]	Japan	2000–2009	678	54.7% (schwannoma)	18.1% (ependymoma)	44.4%/55.6%	52.4
Duong et al., 2012 [3]	USA	1999–2007	11,712	N/A	N/A	59.1%/40.9%	N/A
Ozawa et al., 2013 [7]	Japan	2008–2010	112	53.0% (schwannoma)	18.0% (ependymoma)	46%/54%	55.0
Moein et al., 2013 [9]	Iran	1992–2004	102	45.1% (schwannoma)	35.3% (ependymoma)	42.2/57.8%	40.2
Bansal et al., 2013 [11] ^+^	India	2001–2010	195	0%	100% (ependymoma)	29.7%/70.2%	26.0
Zhixu Ng et al., 2018 [24]	Singapore	2011–2016	91	89.0% (schwannoma)	11.0% (ependymoma)	40.7%/59.3%	46.0
Guerrero-Suarez et al., 2018 [10]	Mexico	2006–2016	35	85.7% (schwannoma)	14.3% (ependymoma)	25.7%/74.3%	43.0
Ouma, 2019 [23] *	South Africa	2014–2017	92	100% (schwannoma)	0%	38.3%/61.7%	N/A
Singuepire et al., 2019 [17]	Cuba	2008–2013	31	42% (N/A)	9.6% (N/A)	54.8%/45.2%	54.4
Tsai et al., 2020 [12]	Taiwan	2004–2014	247	N/A	N/A	50.2%/49.8%	55.4
Brazilian series							
Ferreira et al., 1981 [16]	Brazil	1973–1980	100	N/A	N/A	48%/57%	N/A
Koerbel et al., 2002 [13] ^+^	Brazil	1993–1999	35	0%	100% (ependymoma)	32.4%/68.6%	32.9
Prevedello et al., 2003 [15] *	Brazil	1993–1999	44	100% (schwannoma)	0%	43.2%/56.8%	32.9
Taricco et al., 2008[14] ^+^	Brazil	1992–2005	48	0%	100% (ependymoma)	40%/60%	35.0
Present series	Brazil	1997–2021	104	52.9% (schwannoma)	23.1% (ependymoma)	51%/49%	49.0

Limitations of this study

To the best of our knowledge, this study is the only series of more than 100 cases of primary spinal cord tumors operated at the same institution in Latin America. However, this study had some limitations. As this was an uncontrolled study, associations between outcomes and risk or protective factors were not made. Furthermore, this was a single-center, retrospective study. The quality of the data directly depended on the quality of the medical records, which were not always completed in a complete or standardized way. Moreover, the improvement and worsening rates were not analyzed in relation to the compartment of the tumors. Prospective and larger studies with standardized clinical assessments and with longer follow-up periods are necessary to refine the knowledge about this pathology in this population.

## Conclusions

Among the few available studies on primary spinal cord tumors in the Brazilian population and in other Latin American countries, there was considerable heterogeneity in the inclusion and exclusion criteria. In our series, there was a slight predilection for women, and the predominant histological type was schwannoma. Regarding intramedullary tumors, ependymoma was the most frequent histological type. The clinical outcomes observed in this series suggest that patients tend to benefit from surgical treatment, with greater improvement rates than worsening rates in all analyzed events. Further large populational studies are necessary to elucidate the epidemiology of this entity.

## References

[1] Grimm S, Chamberlain MC (2009). Adult primary spinal cord tumors. Expert Rev Neurother.

[2] Chamberlain MC, Tredway TL (2011). Adult primary intradural spinal cord tumors: a review. Curr Neurol Neurosci Rep.

[3] Duong LM, McCarthy BJ, McLendon RE, Dolecek TA, Kruchko C, Douglas LL, Ajani UA (2012). Descriptive epidemiology of malignant and nonmalignant primary spinal cord, spinal meninges, and cauda equina tumors, United States, 2004-2007. Cancer.

[4] Kumar N, Tan WL, Wei W, Vellayappan BA (2020). An overview of the tumors affecting the spine-inside to out. Neurooncol Pract.

[5] Ottenhausen M, Ntoulias G, Bodhinayake I (2019). Intradural spinal tumors in adults-update on management and outcome. Neurosurg Rev.

[6] Schellinger KA, Propp JM, Villano JL, McCarthy BJ (2008). Descriptive epidemiology of primary spinal cord tumors. J Neurooncol.

[7] Ozawa H, Aizawa T, Kanno H, Sano H, Itoi E (2013). Epidemiology of surgically treated primary spinal cord tumors in Miyagi, Japan. Neuroepidemiology.

[8] Hufana V, Tan JS, Tan KK (2005). Microsurgical treatment for spinal tumours. Singapore Med J.

[9] Moein P, Behnamfar O, Khalighinejad N, Farajzadegan Z, Fard SA, Razavi M, Mahzouni P (2013). A 12-year epidemiologic study on primary spinal cord tumors in Isfahan, Iran. J Res Med Sci.

[10] Guerrero-Suarez PD, Magdaleno-Estrella E, Guerrero-López P, Vargas-Figueroa AI, Martínez-Anda JJ (2018). Intradural spinal tumors: 10 - years surgical experience in a single institution. Clin Neurol Neurosurg.

[11] Bansal S, Ailawadhi P, Suri A (2013). Ten years' experience in the management of spinal intramedullary tumors in a single institution. J Clin Neurosci.

[12] Tsai CY, Tsai TH, Su YF (2020). Surgical treatment of intraspinal tumors in Southern Taiwan: the 30-year experience of a single institution. J Clin Neurosci.

[13] Koerbel A, Tatsui CE, Prevedello DMS (2002). Prognostic factors in the treatment of intramedullary spinal cord tumors. Arq Neuropsiquiatr.

[14] Taricco MA, Guirado VM, Fontes RB, Plese JP (2008). Surgical treatment of primary intramedullary spinal cord tumors in adult patients. Arq Neuropsiquiatr.

[15] Prevedello DM, Koerbel A, Tatsui CE, Truite L, Grande CV, Ditzel LF, Araújo JC (2003). Prognostic factors in the treatment of the intradural extramedullary tumors: a study of 44 cases. Arq Neuropsiquiatr.

[16] Ferreira NP, Chaves DL, Moraes AC, de Oliveira LM (1981). Spinal tumors: apropos of 100 cases. Arq Neuropsiquiatr.

[17] Singuepire A, Acosta HF, Sosa KF (2019). Clinical epidemiological characterization of patients operated on spinal cord tumors. Rev Cuba de Medicina Mil.

[18] Alpízar-Aguirre A, Chávez-Miguel C, Zárate-Kalfópulos B (2009). Tumores intradurales extramedulares primarios tratados en el Instituto Nacional de Rehabilitación. Cir Cir.

[19] Hirano K, Imagama S, Sato K (2012). Primary spinal cord tumors: review of 678 surgically treated patients in Japan. A multicenter study. Eur Spine J.

[20] Gelabert-González M (2007). Primary spinal cord tumours. An analysis of a series of 168 patients. Rev Neurol.

[21] Engelhard HH, Villano JL, Porter KR, Stewart AK, Barua M, Barker FG, Newton HB (2010). Clinical presentation, histology, and treatment in 430 patients with primary tumors of the spinal cord, spinal meninges, or cauda equina. J Neurosurg Spine.

[22] Preston-Martin S (1990). Descriptive epidemiology of primary tumors of the spinal cord and spinal meninges in Los Angeles County, 1972-1985. Neuroepidemiology.

[23] Ouma JR (2019). Intradural extramedullary spinal masses treated at the Wits teaching hospitals between 2014 - 2017.

[24] Ng Z, Ng S, Nga V, Teo K, Lwin S, Ning C, Yeo TT (2018). Intradural spinal tumors-review of postoperative outcomes comparing intramedullary and extramedullary tumors from a Single Institution's Experience. World Neurosurg.

[25] Bhimani AD, Denyer S, Esfahani DR, Zakrzewski J, Aguilar TM, Mehta AI (2018). Surgical complications in intradural extramedullary spinal cord tumors - an ACS-NSQIP analysis of spinal cord level and malignancy. World Neurosurg.

